# Analysis of Residual Stress at the Interface of Epoxy-Resin/Silicon-Wafer Composites During Thermal Aging

**DOI:** 10.3390/polym17010050

**Published:** 2024-12-28

**Authors:** Jianyu Wu, Fangzhou Chen, Jiahao Liu, Rui Chen, Peijiang Liu, Hao Zhao, Zhenbo Zhao

**Affiliations:** Science and Technology on Reliability Physics and Application of Electronic Component Laboratory, China Electronic Product Reliability and Environmental Testing Research Institute, Guangzhou 511370, China; wujianyu@ceprei.com (J.W.); chenfangzhou@ceprei.com (F.C.); liujiahao@ceprei.com (J.L.); chenr@ceprei.com (R.C.); liupeijiang@ceprei.com (P.L.)

**Keywords:** epoxy resin, Raman spectroscopy, residual stress, thermal aging, crack

## Abstract

During the thermal aging process of epoxy resin, microcracks, interfacial delamination, and warpage are the key factors leading to semiconductor device damage. Here, epoxy-resin specimens (EP-Ss) and epoxy-resin/silicon-wafer composites (EP-SWs) were prepared to analyze the distribution of residual stress (RS) in epoxy resin and its thermal aging process changes. The uniaxial tensile approach and Raman spectroscopy (RAS) showed that the peak shift of aliphatic C-O in EP-Ss was negatively correlated with the external stress, and that the stress correlation coefficient was −2.76 × 10^−2^ cm^−1^/MPa. Then, RAS was used to evaluate the RS distribution of EP-SWs, obtaining a high-resolution stress-distribution image of 50 × 50 pixels and revealing a strong stress concentration at the interface between the epoxy resin and the silicon wafer. Additionally, Fourier transform infrared spectroscopy (FTIR), Differential scanning calorimetry (DSC), Field-emission scanning electron microscopy (FE-SEM), and RAS were used to analyze the chemical composition, molecular structure, interfacial microstructure, and RS of the epoxy resin during the thermal aging process. With the increase in the thermal aging time, the epoxy resin underwent secondary curing, the RS at the interface changed from tensile stress to compressive stress, and cracks were formed. The results illuminate the effect of the thermal aging process on the interface-failure mechanism of composite materials, aiding in the reliability evaluation and safety design of semiconductor devices.

## 1. Introduction

Thermosetting materials like epoxy resin are three-dimensional, highly cross-linked networks formed by cross-linking reactions between epoxy-resin monomers or oligomers and curing agents [1,2,3,4]. Epoxy resin has excellent thermal, mechanical, and electrical properties and is widely used in various applications, including adhesives, coatings, electronic packaging, and insulation [5,6,7]. In recent years, the manufacturing of packaging devices, from the simplest plastic encapsulants to advanced packaging in heterogeneous integration, involves a curing process, which inevitably introduces residual stress (RS) [8,9]. Additionally, in a multi-material structure bonded by different materials, owing to the nonuniform chemical and physical shrinkage caused by the thermal aging effect during high-temperature service, large amounts of RS may also be generated. A sufficiently large amount of RS may cause microcracks, interfacial delamination, warpage, or damage to the epoxy resin, which is gradually becoming one of the main challenges affecting the reliability of packaging devices [10,11,12,13,14]. Therefore, evaluating the RS of the material structure is crucial for understanding the impact of thermal aging on the reliability of the composite structure.

In one sense, the initial stress of the epoxy resin-based multi-material structure comes from the thermal RS generated during curing. During the curing process, the epoxy resin shrinks when cooled to room temperature. At this time, the metal or nonmetal substrate has a lower coefficient of thermal expansion and shrinks much less than the epoxy resin, resulting in the generation of RS at the interface between the epoxy resin and the substrate [15,16,17]. In another sense, in a thermal-oxidative environment, the epoxy resin in the multi-material structure undergoes an oxidation reaction, resulting in changes in the density of the polymer (due to the grafting of oxygen atoms along the polymer chain) and mass changes (a large amount of volatile products are formed by a chain breakage near the chain end, ultimately leading to mass loss), finally causing the epoxy resin to undergo volume shrinkage. Owing to the inherent inertness of the substrate, the substrate exhibits negligible deformation at temperatures below 300 °C. Different degrees of oxidative deformation can be observed between the shrinking epoxy-resin matrix and the substrate, thus changing the RS at the interface [18,19,20]. With the increase in the duration of the thermal aging process, the RS at the interface further accumulates, causing warpage and interfacial peeling of the material structure products [21]. Therefore, understanding the evolution law of the RS during the thermal aging process is an important research topic for exploring the correlation between thermal aging and structural damage of the composite material.

Currently, many techniques are available for characterizing residual stress, including X-ray diffraction (XRD), Raman spectroscopy (RAS), ultrasonic acoustics, and strain gauges [16]. Compared to ultrasonic acoustics and strain gauges, which can only obtain residual stresses in large areas, XRD and Raman spectroscopy can accurately measure residual stresses in micro areas. However, X-ray diffraction is limited to residual stress measurement of materials with crystallinity and is not suitable for amorphous materials such as epoxy resins. Therefore, RAS is currently an important multi-scale and nondestructive approach for characterizing the RS of amorphous polymer-based materials with high resolution. Based on the change in vibrational energy in polymer molecules under mechanical stress, RAS can confirm the displacement value of sensitive bands and then calculate the RS, so it has been widely used to understand the mechanical properties of various polymers [22,23,24,25]. Abiko et al. employed RAS to evaluate the RS distribution of epoxy-resin/aluminum composites [23]. Through Raman imaging, it was found that compared with room-temperature curing, a more intense stress concentration was found at the interface between the epoxy resin and the aluminum. Wu et al. imaged the RS of an epoxy-based thermosetting material by RAS and found that the RS of the thermosetting material containing a highly dynamic thiocarbamate bond decreased by 44% after annealing at 30 °C [24]. These studies have shown that the RS caused by differences in the curing process and physical properties of the resin of the polymer-based material can be detected and analyzed by Raman imaging. Therefore, Raman imaging is beneficial for better understanding the evolution of the micromechanics and complex failure processes of the composite structure, including interfacial debonding, matrix cracking, warpage, and local stress distribution. However, its related applications in the thermal aging of polymer-based materials have been rarely reported.

Here, epoxy-resin specimens (EP-Ss) and epoxy-resin silicon-wafer composites (EP-SWs) were prepared. The uniaxial tensile approach and RAS were used to study the relationship between the stress and peak shift of the EP-Ss. Then, the EP-SWs were placed in an oven for thermal aging test at 105 °C. The microscopic RS distribution of the EP-SWs during the thermal aging process at different times was measured. The distribution uniformity and evolution law of the RS at the interface of the composite structure were analyzed. Additionally, Fourier transform infrared spectroscopy (FTIR), Differential scanning calorimetry (DSC), and Field-emission scanning electron microscopy (FE-SEM) were used to analyze the chemical composition, molecular structure, and interfacial microstructure of the epoxy resin after the thermal aging process.

## 2. Materials and Methods

### 2.1. Materials

The bisphenol-A diglycidyl, 4,4′-methylenedianiline, and N,N-dimethylformamide used in this work were all purchased from Shanghai Aladdin Biochemical Technology Co., Ltd. (Shanghai, China). The silicon wafers were purchased from Shijiazhuang Jing Yan Electronic Technology Co., LTD (Shijiazhuang, China). All the above samples were used as received, without purification treatment.

### 2.2. Sample Preparation

#### 2.2.1. Preparation of Epoxy-Resin Specimens (EP-Ss)

The EP-Ss for uniaxial tensile approach were prepared as follows. The bisphenol-A diglycidyl ether monomer and 4,4′-methylenedianiline curing agent were dissolved in a flask containing 2 mL of N,N-dimethylformamide solution at a weight ratio of 4.5:1 to obtain a mixed solution. The mixed solution was then cast in a rectangular mold (L80 × W5 × T2 mm) and cured in a vacuum at 60 °C for 24 h to obtained EP-Ss.

#### 2.2.2. Preprocessing of Silicon Wafers

Silicon wafers measuring 10 mm × 10 mm × 0.6 mm were transferred to a beaker containing 30 mL of chloroform and ultrasonically cleaned for 30 min. Then, the silicon wafer was transferred to a plasma cleaner for atmospheric-pressure treatment for 5 min.

#### 2.2.3. Preparation of EP-SWs

First, the bisphenol-A diglycidyl ether monomer and 4,4′-methylenedianiline curing agent were dissolved in a flask containing 5 mL of N,N-dimethylformamide solution at a weight ratio of 4.5:1. The mixed solution was mechanically stirred in a vacuum environment at 60 °C for 30 min to remove bubbles, and the DMF solution was added to the mixed solution, thereby preparing a prepolymer solution. Then, the prepolymer solution was poured into a silicone mold with a silicon wafer attached to the bottom and cured in a vacuum at 80 °C for 8 h and at 100 °C for 2 h. After curing, the heating was stopped, and the temperature in the oven was slowly cooled to room temperature. After demolding, EP-SWs were obtained by cutting and polishing. All samples were cuboids of 10 mm in length, 10 mm in width, and 5 mm in height.

### 2.3. Thermal Aging Process

EP-SWs were isothermally oxidized and aged in an air-circulating oven, which could continuously deliver external air to the oven to maintain a fresh oxygen environment around the EP-SWs. To exclude the moisture effect, all the specimens were baked in an oven at 60 °C for 2 h. Subsequently, under high-temperature atmosphere conditions, the cured EP-SWs were placed in the oven. The thermal aging was conducted based on the standard IEC 60505 using an oven with a stable temperature of 105 °C [26]. The specimens were taken out regularly, and the time span was 2, 6, 14, and 25 d, respectively.

### 2.4. Characterization and Measurement

#### 2.4.1. RS Measurement

##### Chemical Structure Analysis

To analyze the changes in chemical bonds of the epoxy resin during curing and thermal aging, we carried out a Fourier transform infrared spectroscopy measurement in the attenuated total reflection (ATR) mode (Nicolet IS50 + Continuum, Thermo Fisher Scientific Co., Ltd., Waltham, MA, USA) with 16 accumulative scans, a resolution of 2 cm^−1^, and a scanning range of 500–4000 cm^−1^. Additionally, the analysis process of the infrared spectrum was carried out using Spectrum software (PerkinElmer, Glen Waverley, VIC, Australia).

##### Glass Transition Measurement

A differential scanning calorimetry measurement was carried out using a DSC Q1000 (METTLER TOLEDO, Switzerland). The sample with a mass range of 3–5 mg was sealed in an aluminum pan and heated from 0 to 250 °C at a ramp rate of 10 °C/min under a nitrogen flow of 50 mL/min. The results were interpreted using TA analysis software. DSC analysis was carried out to check the total curing degree of the sample and measure the glass transition temperature value of the aged sample. The Tg was measured during the second heating ramp at the inflection point of the thermogram (i.e., after removing the thermal history of the sample).

##### Micromorphology Characterization

Before imaging the microregion, the sample measurement area was sprayed with platinum via an ion sputtering coating to improve the resolution. An Apreo 2 field-emission scanning electron microscope produced by Thermo Fisher Scientific was used to observe the microscopic morphology of the EP-SW.

## 3. Results and Discussion

### 3.1. Relationship Between Peak Shift and Stress

**Figure 1:**
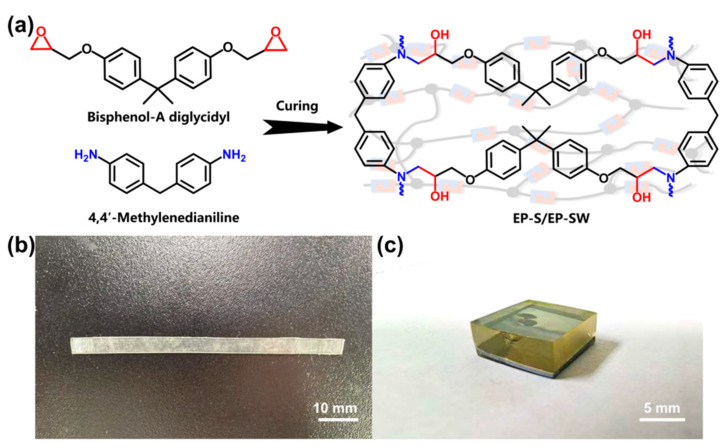
(**a**) Schematic illustration of preparation of the epoxy resin. (**b**) Image of EP-S. (**c**) Image of EP-SW.

**Figure 2:**
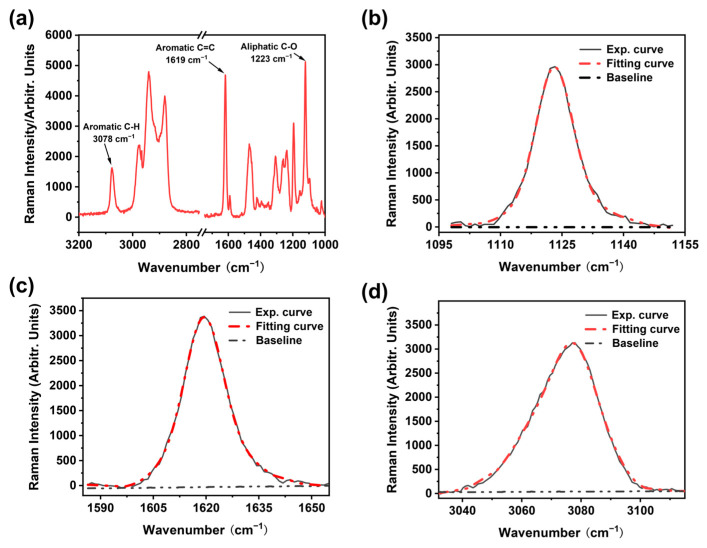
(**a**) Raman spectra of epoxy resin; (**b**) 1123 cm^−1^ (aliphatic C-O stretching vibration) peak and the results fit of the data; (**c**) 1619 cm^−1^ peak (aromatic C=C stretching vibration) and the results fit of the data; (**d**) 3078 cm^−1^ peak (aromatic C-H stretching vibration) and the results fit of the data.

As shown in Figure 1, EP-Ss and EP-SWs were prepared by a nucleophilic addition reaction, using bisphenol-A diglycidyl ether as the matrix and 4,4′-methylenedianiline as the curing agent. The Raman spectrum of the EP-Ss in the range of 1000–3200 cm^−1^ is shown in Figure 2a. Since the narrower the width of the Raman peak, the greater the improvement in the measurement accuracy of the peak shift, combined with the stress measurement approaches reported in the existing literature, the peaks at 3078 cm^−1^, 1618 cm^−1^, and 1123 cm^−1^ with narrower half-width peaks were selected for stress measurement and analysis. These three peaks correspond to the aromatic C-H stretching vibration, aromatic C=C stretching vibration, and aliphatic C-O stretching vibration, respectively. After continuously collecting the Raman spectra at the same point, we finally obtained the accurate peak positions by curve fitting using the Voigt function (Gaussian + Lorentzian function), as shown in Figure 2b–d.

**Figure 3:**
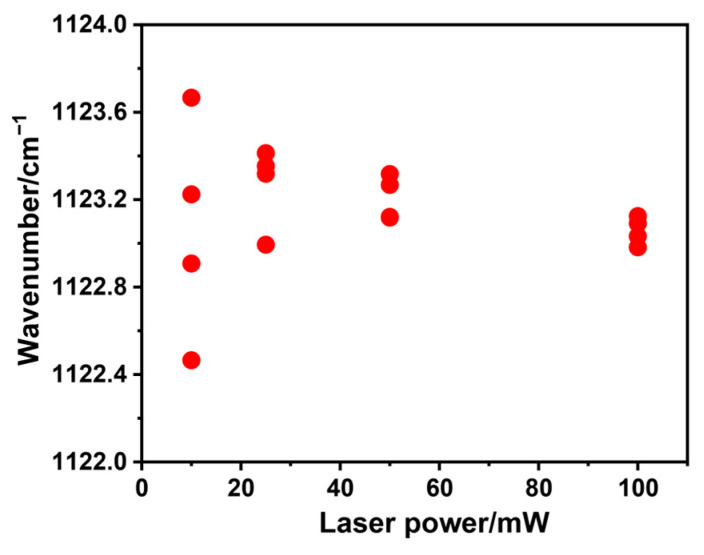
Peak position as a function of illumination laser power.

To confirm the influence of the laser-irradiation power intensity on the Raman peak position, we measured the EP-Ss with different laser powers and fitted the peak positions, as shown in Figure 3. It could be observed that, owing to the local temperature rise in the measurement area caused by laser irradiation, the peak position decreased with the increase in the laser power. However, as long as the laser power was constant, this thermal shift was expected to be constant. Additionally, with the increase in the laser power, the intensity of the Raman characteristic peak also increased, and the deviation of the peak position obtained by Voigt fitting was smaller. Therefore, to ensure the reproducibility and accuracy of the measurement data, we used the maximum laser power for measurement in this work.

**Figure 4:**
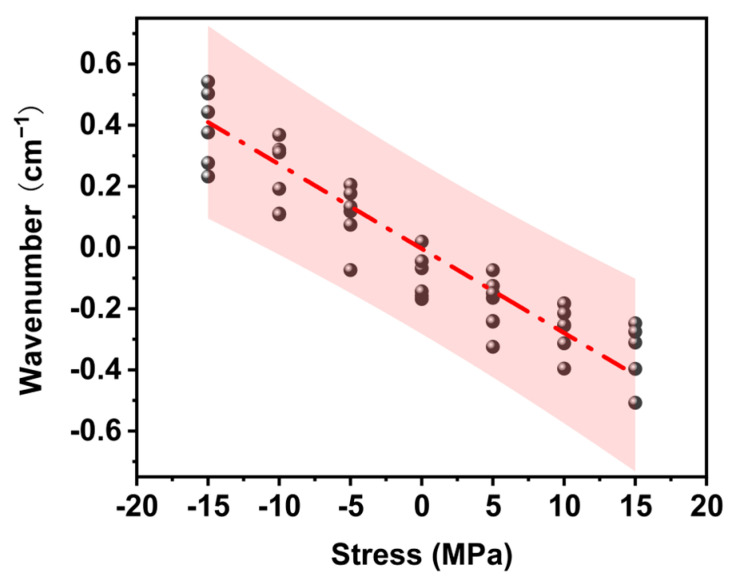
The straight line of the relationship between the frequency shift of the 1223 cm^−1^ peak of the epoxy resin and the stress caused by the load in the uniaxial tensile approach represents the least-square fitting regression line of the data points.

Based on the ability of RAS to detect the local change in molecular vibration energy, by measuring the corresponding relationship between strain and Raman wave number and combining the linear relationship between the stress and strain of elastic mechanical materials, researchers can obtain the Raman frequency shift–stress factor, and the relationship between the material stress and Raman frequency shift can be established. It has been experimentally demonstrated that when using RAS to measure the stress of amorphous materials, according to Hooke’s law, the relationship between the peak shift and stress is shown in Equation (1) [22]:

(1) $$∆v=v_{\sigma }-v_{0}=S^{\sigma }\times ∆\sigma ,$$

where ∆ν represents the peak shift; ∆σ represents the stress; ν_σ_ and ν_0_ are the peak positions with and without applied stress, respectively; and S^σ^ is the Raman mechanical coefficient. In the uniaxial tensile approach, a small uniaxial tensile platform is used to apply tensile load to the epoxy resin, and the laser is focused on the sample surface to obtain the Raman spectrum. Figure 4 shows the peak shift diagram of EP-Ss. The peak shift reflected the difference in the relative peak positions before and after the application of stress. The absolute value of the peak shift decreased linearly with the increase in the load. When an external compressive stress load was applied, owing to the shortening of the molecular bond length, the vibration frequency increased, and the spectral band shifted in the high-frequency direction (the wave number becomes larger); conversely, when the epoxy resin was subjected to a tensile stress load, the spectral band shifted in the low-frequency direction (the wave number became smaller). A regression line was obtained by the least square approach, and all the data points were located within the 95% prediction interval.

**Figure 5:**
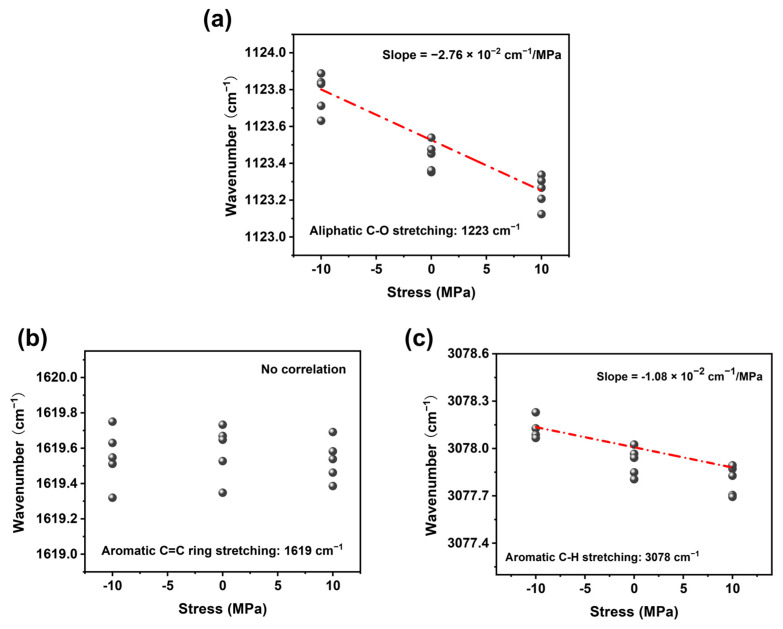
(**a**) Raman frequency shift–stress relationship diagram of the 1223 cm^−1^ aliphatic C-O stretching vibration peak. (**b**) Raman frequency shift–stress relationship diagram of the 1619 cm^−1^ aliphatic C=C stretching vibration peak. (**c**) Raman frequency shift–stress relationship diagram of the 3078 cm^−1^ aliphatic C-H stretching vibration peak.

From the slope of the regression line, the Raman mechanical coefficient of the 1123 cm^−1^ peak was −2.76 × 10^−2^ cm^−1^/MPa (Figure 5a). Additionally, the stress-peak shift detection was also carried out for the stretching vibration of aromatic C=C at 1619 cm^−1^ and aromatic C-H at 3078 cm^−1^. The aliphatic C=C peak did not show stress sensitivity associated with stress (Figure 5b). The Raman mechanical coefficient of the 3078 cm^−1^ peak was −1.08 × 10^−2^ cm^−1^/MPa. In the literature, the Raman mechanical coefficient has been reported at −1.00 × 10^−2^ cm^−1^ /MPa of the stretching vibrations of aromatic C-H of epoxy resin. The difference in constants is due to the different modes of vibration produced by the protons in the aromatic ring or the aromatic ring skeleton. By observing the stretching vibrations of aromatic C-H and aliphatic C-O, it can be seen that the Raman stress correlation coefficients were all negative, which is in line with the corresponding relationship between the measured strain and Raman wave number (Figure 5c). Comparing the stress coefficients of aromatic C-H and aliphatic C-O, we found that the stress sensitivity of aromatic C-H was less than half, indicating that the aliphatic C-O peak can be used for stress measurement of the epoxy resin and can provide more accurate data measurement results. Combining the above results, aliphatic C-O was selected as the Raman peak for stress measurement in this work.

### 3.2. Imaging of RS Distribution

**Figure 6:**
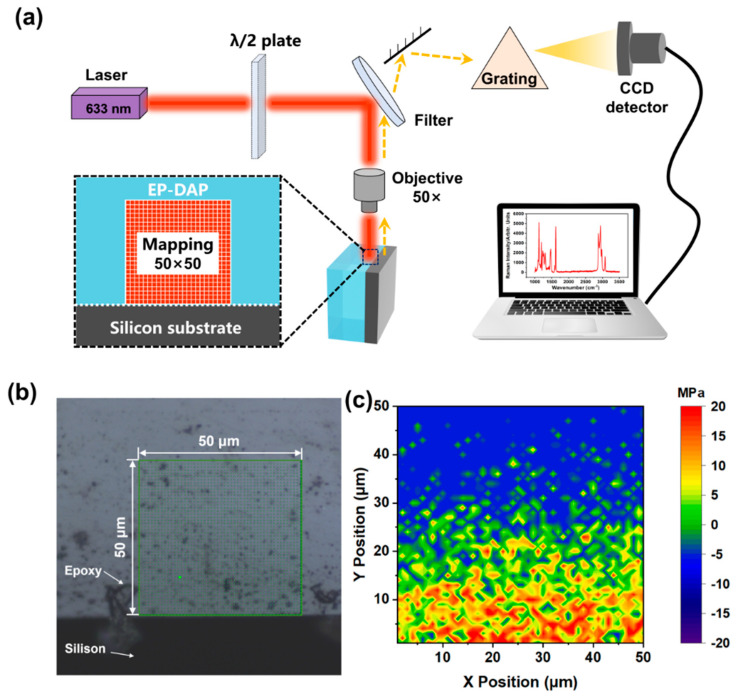
(**a**) Schematic diagram of the residual stress (RS) testing process. (**b**) Image of the Raman spectroscopy measurement area. (**c**) Image of RS distribution of epoxy resin at the interface.

RAS was used to measure the RS of the sample after curing. The schematic diagram of the Raman imaging test process of RS and measurement area are shown in Figure 6a,b. The X-axis was defined as the distance parallel to the bonding interface. The Y-axis was defined as the vertical distance from the EP-SW interface. The same Raman mechanical coefficient (−2.76×10^−2^ cm^−1^/MPa) was used to measure the RS of the unaged epoxy resin. Then, the measurement area was characterized by RAS with a scanning step size of 1 μm. By measuring the 1123 cm^−1^ peak shift and calculating the RS of each point, the distribution image of RS in the region of 50 × 50 μm^2^ could be obtained, as shown in Figure 6c. Compared with the literature, the distribution of residual stress in epoxy resin shows similar result [23]. Furthermore, it can be seen that at the interface (X = 0), a large tensile stress (11.64 Mpa) of the epoxy resin was detected because the epoxy resin matrix was restrained by the silicon wafer. As the distance between the measuring position and the interface increased, the residual stress decreased gradually. Interestingly, at a distance of 25 microns from the interface, the epoxy resin showed zero stress, which was caused by matrix shrinkage and silicon chip restraint. When the measurement position continues to increase, the matrix shrinkage occupies the dominant factor and presents compressive stress (5.40 Mpa).

### 3.3. Thermal Aging Analysis

**Figure 7:**
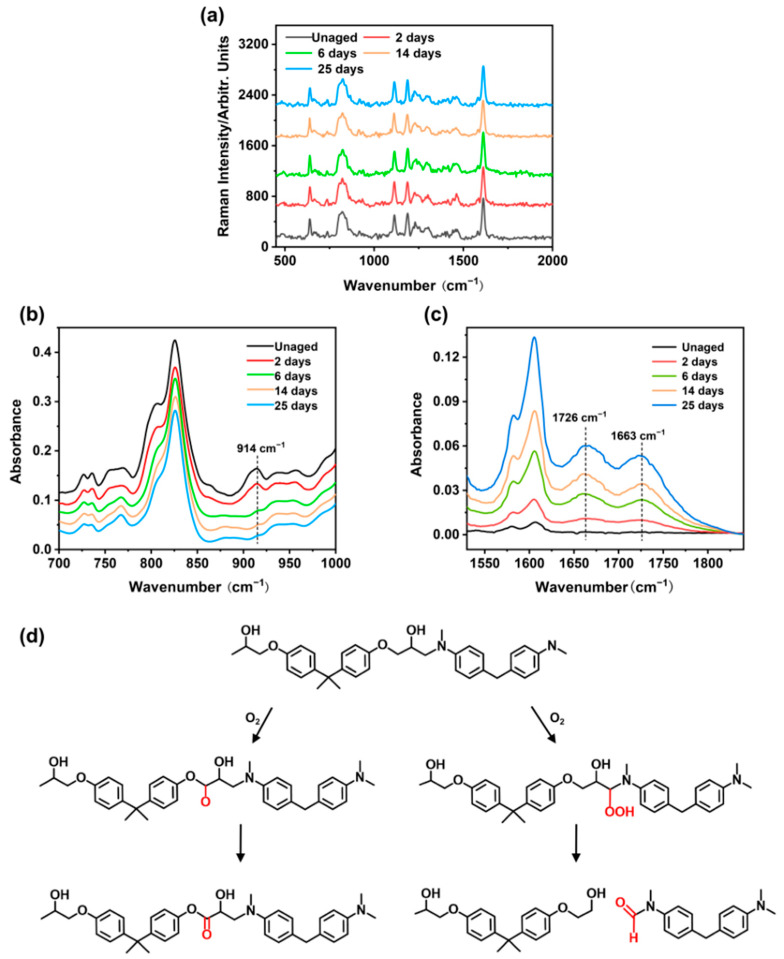
(**a**) Raman spectra of EP-SWs with different aging times in the wavenumber range of 450–2000 cm^−1^. (**b**) FTIR spectra of EP-SWs with different aging times in the wavenumber range of 700–1000 cm^−1^. (**c**) FTIR spectra of EP-SWs with different aging times in the wavenumber range of 1570–1860 cm^−1^. (**d**) Schematic diagram of thermal degradation mechanism of epoxy resin.

Under dry conditions, the cured EP-SW was exposed at 105 °C for 25 days and detected regularly using RAS and ATR-FTIR, respectively. As shown in Figure 7a, the characteristic peaks of epoxy resin at different aging times are basically consistent, which does not prove that the epoxy resin undergoes thermal degradation reaction. This phenomenon may be due to the fact that thermal degradation of polymer produced polar groups, whereas RAS was only sensitive to non-polar groups in the molecular chain. To demonstrate that the polymer undergone thermal degradation during thermal aging, ATR-FTIR was used to characterize the epoxy resin. In Figure 7b, the 914 cm^−1^ band corresponded to the epoxy group, and the peak height of the band gradually decreased and disappeared with the extension of high-temperature loading time, which could be attributed to the secondary cross-linking of the epoxy resin during the thermal aging process. As shown in Figure 7c, the detection of the epoxy amine infrared fingerprint region showed that the main changes caused by thermal oxidation occurred in two peaks within the wave number range of 1570–1860 cm^−1^. The peak at 1663 cm^−1^ usually came from the amide group created by aging on the molecular chain. The band at 1726 cm^−1^ usually came from the vibration of the carbonyl group produced by auto-oxidation [27]. The schematic diagram of the thermal degradation mechanism of epoxy resin is shown in Figure 7d. The growth of both bands was referred to the thermo-oxidative degradation of the epoxy chains.

**Figure 8:**
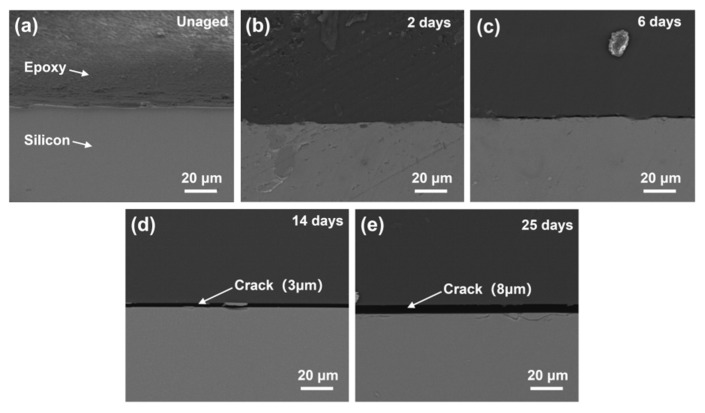
FE-SEM images of (**a**) unaged, (**b**) aged for 2 days, (**c**) aged for 6 days, (**d**) aged for 14 days, and (**e**) aged for 25 days.

The interface position between epoxy resin and silicon wafer with different thermal aging times was observed using scanning electron microscopy. Before thermal aging, the epoxy resin at the interface was fully bonded to the silicon wafer without any cracks, indicating the formation of a strong and stable composite structure. When the sample was baked at 105 °C for 14 days, although the interface was still relatively flat, a small crack appeared at the interface. This phenomenon indicated that RS within the matrix have an impact on the morphology of the interface. After 6 days of aging, a crack with a width of 3 μm was formed at the interface position, as shown in Figure 8. This might be due to the increase in the cross-linking density of the epoxy-resin matrix during secondary curing, resulting in shrinkage stress at the interface, making the epoxy resin unable to withstand high stress and peel off to form cracks. After 25 days, the crack significantly expanded, indicating that the RS of the material continuously accumulated and led to the further expansion of the crack.

**Figure 9:**
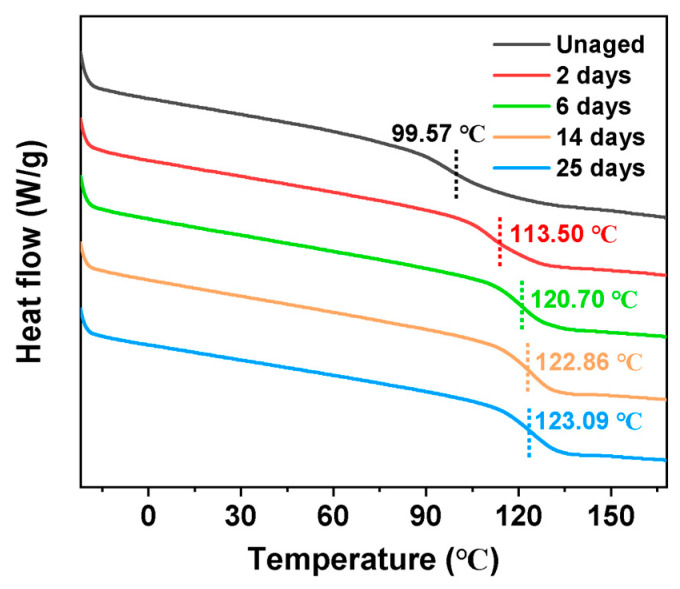
DSC curves of epoxy-resin samples at different aging times during the thermal aging process at 105 °C.

DSC curves of different aging times are shown in Figure 9. Endothermic reactions were observed in EP-SWs both before and after thermal aging over a wide temperature range, and differences existed between the glass transition temperatures of aged and nonaged epoxies. The obvious glass transition temperatures (Tgs) were 99.57 °C, 113.50 °C, 120.70 °C, 122.86 °C, and 123.09 °C for the samples that were not aged, aged for 2 d, aged for 6 d, aged for 14 d, and aged for 25 d, respectively. It can be observed that the Tg of the whole system gradually increased with the increase in aging time, and that the Tg value tended to be stable after 14 d of aging, which is considered to be the result of the joint action of cross-linking and chain-breaking effect of epoxy resin [28].

**Figure 10:**
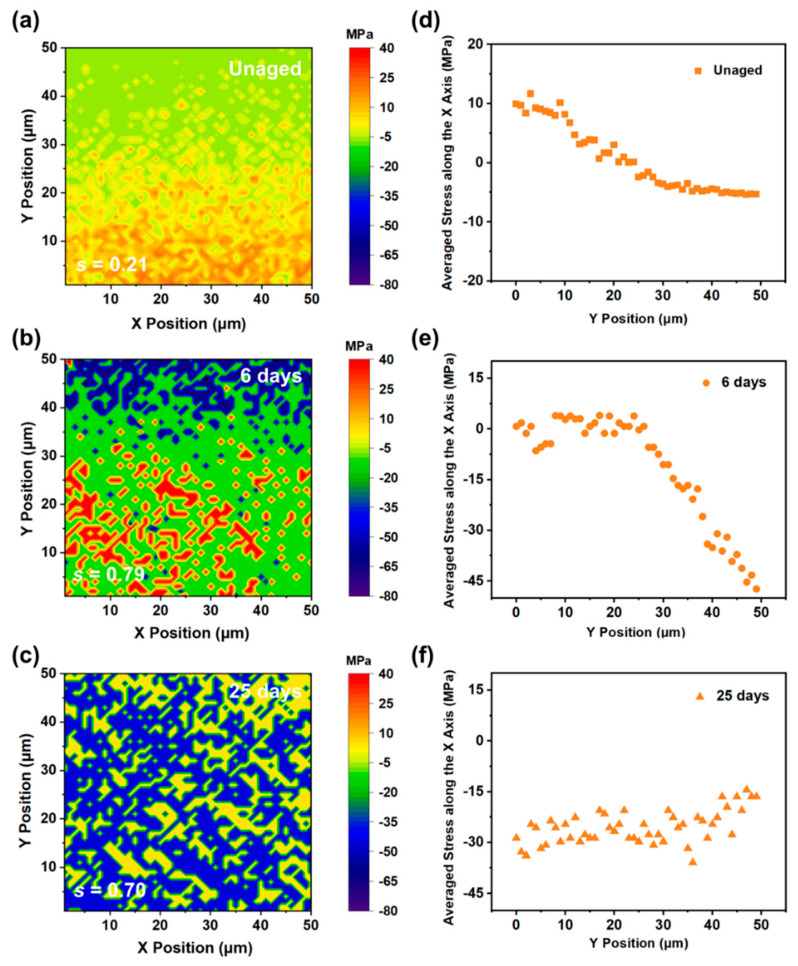
(**a**) Distribution of RS at the interface of unaged (EP-SW). (**b**) Distribution of RS at the interface of EP-SW aged for 6 days. (**c**) Distribution of RS at the interface of EP-SW aged for 25 days. (**d**) Average stress along the X-axis of the interface of unaged epoxy resin obtained by Raman imaging. (**e**) Average stress along the X-axis of the interface of epoxy resin aged for 2 days obtained by Raman imaging. (**f**) Average stress along the X-axis of the interface of epoxy resin aged for 25 days.

To verify the evolution law of the interfacial RS, we selected cross sections of the unaged, aged for 6 d, and aged for 25 d EP-SWs for Raman imaging. Figure 10 shows the distribution of the RS of the epoxy resin near the position of the silicon wafer. According to the results of the Raman imaging, owing to the mismatch of the thermal expansion coefficients between the epoxy resin and the silicon wafer, the unaged EP-SWs formed tensile stress at the interface, and this stress state changed to compressive stress as the distance from the interface increased. With the increase in the thermal aging time, owing to the increase in the cross-linking density of the matrix caused by the secondary curing inside the epoxy resin, the shrinkage stress at the interface was further increased. Notably, after 25 d of aging, owing to the cracks formed at the epoxy-resin/silicon-wafer interface, the matrix was no longer constrained by the silicon wafer at all, and the entire area uniformly presented a shrinkage stress.

Additionally, the parameter s, which represents the standard deviation of the sample, was used to quantify the uniformity of the RS distribution, as shown in Equation (2):

(2) $$s^{2}=\frac{\sum_{i=1}^{n}{\left(x_{i}-x\right)}^{2}}{n-1}$$

where s^2^ is the sample variance, n is the number of test points, x_i_ is the fitted value of the Raman shift at the i-th point, and x is the average value. Before aging, since the cross-linking curing inside the epoxy resin was not yet complete, the distribution of tensile stress at the interface was not obvious, and the value of s was 0.21. After aging at 105 °C for 6 d and 25 d, the epoxy resin showed significant stress differences owing to the peeling off of the EP-SW adhesive interface, resulting in matrix shrinkage and molecular chain fracture with values of 0.79 and 0.70, respectively.

To compare the stress change trends in the depth direction of the interfaces (Y-axis) of different aging times of EP-SW, we averaged the relative stresses on the surfaces of the epoxy resins in the X-axis direction. As shown in Figure 10d, the RS increased as the distance to the silicon interface decreased, and a tensile stress of 9.9 MPa was detected on the resin surface. With the increase in the time of the thermal aging process, the RS at the interface further accumulated, and the shrinkage stress formed inside the matrix gradually intensified. Finally, in the epoxy resins shown in Figure 10e,f, the average shrinkage stress on the resin surface of the entire area was approximately 25.5 MPa. The above results further confirm that the formation and expansion of cracks was caused by the increase in the shrinkage stress inside the matrix.

## 4. Conclusions

In this paper, we prepared epoxy-resin/silicon-wafer composites with a bilayer structure. The RS of the epoxy-resin/silicon-wafer composites during the curing and thermal aging process was measured with a Raman spectrometer. The distribution of the RS on the cross section of the epoxy-resin/silicon-wafer composites was measured and calculated, and two-dimensional RS images on the epoxy-resin interface were acquired. The stress images clearly show that, stress concentration occurred near the interface of the resin/silicon wafer composites after curing process, because the thermal contraction amplitude of the epoxy resin was greater than that of the silicon wafer when cooling to room temperature after molding. As the exposure time in the thermal aging environment at 105 °C continued to increase, the stress at the interface gradually transformed into shrinkage stress and gradually increased, eventually leading to the formation of cracks. Meanwhile, FTIR was used to analyze the molecular structure of the epoxy resin after thermal aging. The results show that with the increase in the thermal aging time, secondary curing occurred in the epoxy resin, the RS at the interface changed from tensile stress to compressive stress, and cracks were formed. Therefore, in this work, research on the characterization approach of micro-region RS was carried out by combining Raman spectroscopy, revealing the distribution state of the interfacial RS. Additionally, the development of the reliability-evaluation technology for RS and the clarification of the correlation between the evolution of RS and its crack initiation have important application research value and practical significance for evaluating the technology of local thermal RS at the interface of multi-material structures.

## Data Availability

The original contributions presented in the study are included in the article, and further inquiries can be directed to the corresponding authors.
